# Head and Trunk Kinematics during Activities of Daily Living with and without Mechanical Restriction of Cervical Motion

**DOI:** 10.3390/s22083071

**Published:** 2022-04-16

**Authors:** Angela R. Weston, Brian J. Loyd, Carolyn Taylor, Carrie Hoppes, Leland E. Dibble

**Affiliations:** 1 Department of Physical Therapy and Athletic Training, University of Utah, 520 Wakara Way, Salt Lake City, UT 84108, USA; lee.dibble@hsc.utah.edu; 2 Department of Physical Therapy and Rehabilitation Sciences, University of Montana, 32 Campus Dr., Missoula, MT 59812, USA; brian.loyd@mso.umt.edu; 3 Department of Orthopedics, University of Utah, 590 Wakara Way, Salt Lake City, UT 84108, USA; carolyn.taylor@utah.edu; 4 Army Baylor University Doctoral Program in Physical Therapy, U.S. Army Medical Center of Excellence, 3630 Stanley Road, San Antonio, TX 78234, USA; carrie.w.hoppes.mil@army.mil

**Keywords:** wearable sensors, turning, activities of daily living, head–trunk kinematics

## Abstract

Alterations in head and trunk kinematics during activities of daily living can be difficult to recognize and quantify with visual observation. Incorporating wearable sensors allows for accurate and measurable assessment of movement. The aim of this study was to determine the ability of wearable sensors and data processing algorithms to discern motion restrictions during activities of daily living. Accelerometer data was collected with wearable sensors from 10 healthy adults (age 39.5 ± 12.47) as they performed daily living simulated tasks: coin pick up (pitch plane task), don/doff jacket (yaw plane task), self-paced community ambulation task [CAT] (pitch and yaw plane task) without and with a rigid cervical collar. Paired *t*-tests were used to discern differences between non-restricted (no collared) performance and restricted (collared) performance of tasks. Significant differences in head rotational velocity (jacket *p* = 0.03, CAT-pitch *p* < 0.001, CAT-yaw *p* < 0.001), head rotational amplitude (coin *p* = 0.03, CAT-pitch *p* < 0.001, CAT-yaw *p* < 0.001), trunk rotational amplitude (jacket *p* = 0.01, CAT-yaw *p* = 0.005), and head–trunk coupling (jacket *p* = 0.007, CAT-yaw *p* = 0.003) were captured by wearable sensors between the two conditions. Alterations in turning movement were detected at the head and trunk during daily living tasks. These results support the ecological validity of using wearable sensors to quantify movement alterations during real-world scenarios.

## 1. Introduction

Successful participation in daily life activities are critical components of a person’s functional status and should be the goals for rehabilitative interventions. To understand limitations experienced by an individual with a health condition, rehabilitation professionals rely on varied types of functional assessments typically performed in constrained clinical or laboratory settings. They provide insight into a person’s functional status; however, accepting the presumption that functional assessments are replicating the necessary mobility, coordination, and strength required to complete activities of daily living (ADL) [1] would be overlooking a large part of patients’ function.

Another major limitation to clinic-based functional assessments is the tendency for patients to perform at a higher level during clinic or laboratory assessments than during normal daily activity [2,3]. These studies suggest that patients’ performance may be affected by observation, masking their true representative mobility throughout the day. Rating a patient based on their laboratory assessment may give insight to their mobility capacity but may not represent the strategies a patient chooses during ADL. Standardized functional assessments often rely on restrained scoring criteria [4,5,6], do not measure how a patient truly performs daily life activities, and are still vulnerable to inaccuracies [7,8]. Inertial measurement units (IMUs) are a recently available tool that may be able to measure differences in movements that are not detected through visual observation or clinical scales. For example, IMUs detected significant differences in postural stability and functional assessment performance between healthy controls and people with mild impairments when standardized assessments were unable to discern between the groups [7,9,10,11].

Head turning and head–trunk coupling metrics have been of interest in clinical populations [12,13,14,15]. Limiting head motion and alteration of head and trunk coupling is often seen to minimize the provocation of symptoms such as dizziness and unsteadiness [14,15]. Head turn metrics captured by IMUs allow for quantification of head kinematic differences found between individuals with vestibular hypofunction and controls [13,16], as well as between those with mild traumatic brain injury and controls [10]. Evaluating individuals with vestibular hypofunction or sensory integration deficits during ADL tasks allows for a more realistic assessment of head and trunk kinematics than clinic based visual observation. For example, during ADL tasks, head turn metrics were altered in a vestibular hypofunction population compared to healthy controls [17].

It would be unreasonable to expect clinicians to be able to visually recognize subtle and complex metrics captured with wearable technology such as IMUs. IMUs have demonstrated sensitivity in capturing subtle movement disruptions that are important clinical differences [10,18,19]. They are reliable and accurate in quantifying head and trunk control [20,21,22,23] and sensitive in identifying physically induced restriction [24]. IMU-based measurements possess ecological utility (i.e., usefulness in real world natural scenarios that can characterize clinical populations during ADL). Given the evidence that wearable sensors provide clinically relevant information that can improve rehabilitation, additional work is necessary to validate the tasks and metrics that reflect daily life function. This brief report sought to determine if IMUs and our data processing algorithms were capable of discerning differences in head and trunk rotation during ADL with a physically-induced restriction of range of motion. We hypothesized rotational velocity, amplitude, and head and trunk coupling would differ between an unrestricted control condition and a mechanical restriction of the neck during ADL.

## 2. Materials and Methods

### 2.1. Study Participants

Based on the large effect sizes for head rotation velocity (Cohen *d* of 1.3–2.0) between vestibular hypofunction patients and healthy controls [13], our power analysis revealed a sample size of 10 participants would have 95% power to detect a difference between groups on our primary outcome. Study participants were a subset of healthy adults between 19 and 55 years old, able to walk without assistance, who were voluntarily recruited as controls for a larger study. Participants were excluded if they had sustained a lower extremity injury within the past 12 months or had any neurological or vestibular conditions. Written informed consent was obtained from all participants prior to data collection. This study was approved by the University of Utah Institutional Review Board.

### 2.2. Experimental Protocol

**Figure 1:**
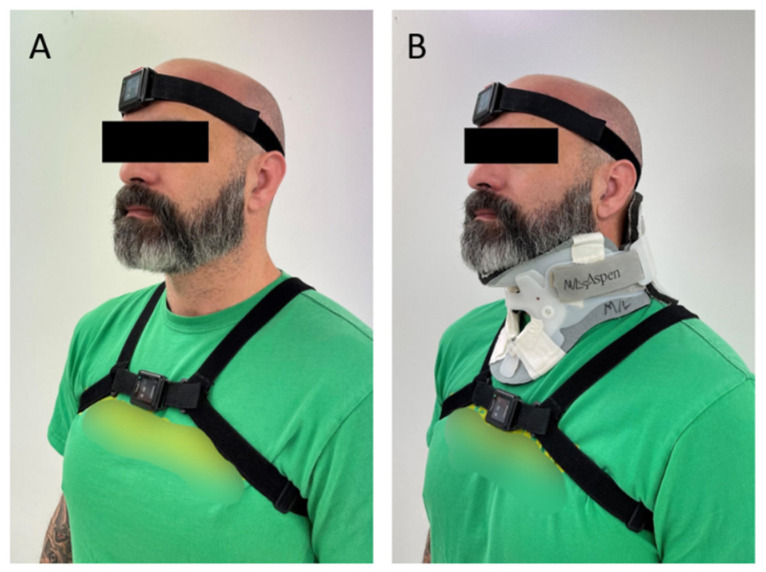
(**A**) Participant fitted with IMUs on forehead and sternum. (**B**) Participant fitted with IMUs and rigid cervical collar.

Ten participants performed tasks twice in a standardized order during one day of testing, once without restriction (our control condition) and again with a rigid cervical collar (Aspen Medical Products, Irvine, CA, USA) to limit cervical motion (Figure 1). The order of task was not randomized as these tasks were part of a larger study protocol and selected for this analysis for their rotational component in the yaw or pitch planes. Data were collected simultaneously by two Opal inertial sensors (APDM Inc., Portland, OR, USA) worn by participants on the forehead and sternum (Figure 1). The location of IMUs was consistent, and elastic straps were adjusted for a secure fit to avoid excessive motion and ensure that the IMUs did not restrict the participant’s movement. The IMUs recorded the participants performing three tasks chosen to represent ADL that required head and trunk rotational motions that are known to be challenging for individuals with vestibular or sensory integration deficits: don/doff a jacket, coin pick up, and a community ambulation task (CAT). To allow for natural performance, directions for tasks were kept simple, and researchers avoided specific instruction on how to perform tasks.

#### 2.2.1. Don/Doff Jacket

The don/doff jacket task began with the participant in a standing position, a jacket placed on a table to their left, and a researcher standing to their right. When instructed, the participant reached for the jacket, donned and doffed the jacket, and returned the jacket to the researcher. Instructions for participants were, “pick up the jacket on your left, put it on, take it off, and hand it to the person on your right”. This task was selected for its required movement in the yaw plane or the anatomical transverse plane.

#### 2.2.2. Pick Up Coin

The coin task began with the participant and researcher facing each other in a standing position and a coin placed on the ground about 12 inches directly in front of the participant. When instructed, the participant bent forward, picked up the coin and placed it in the hand of the researcher. Instructions for participants were, “pick up the coin on the floor in front of you, and place it in the hand of the person in front of you”. This task was selected for its required movement in the pitch plane or the anatomical sagittal plane.

#### 2.2.3. The Community Ambulation Task (CAT)

The CAT consisted of an established 10-min walking route through a university building and the surrounding area. Participants performed the CAT at a self-selected pace while negotiating hallways, stairwells, elevators, and navigating pedestrian and vehicular traffic. A research staff member accompanied the participant and provided landmark-based directions to guide participants throughout the route. At predetermined locations in the route, the staff member asked questions about the surrounding environment that resulted in the participant performing yaw and pitch place head rotations (e.g., what is the name of the room on your right; what color is the tape on the floor). The purpose of the CAT was to mimic a real-world scenario that included both body turns and head turns in the yaw and pitch planes.

### 2.3. Data Acquisition and Outcomes

Wearable IMUs (Opal Wearable Sensors, APDM Inc., Portland, OR, USA) with on-board accelerometers, gyroscopes, and magnetometers were utilized to track head and trunk movement characteristics. Following data collection, gyroscopic data reflecting angular motion of the head and trunk were exported and processed using a custom MATLAB (Mathworks, Natick, MA, USA) algorithm. This processing involved registration of all three sensors to a global reference frame aligned with the *z*-axis to gravity to ensure planar motion was consistent regardless of the angle of the sensor. Processing sensor data in the global reference frame allowed measurement of head and trunk kinematics independent of the anatomical variability in participants. In the event that measuring head and trunk kinematics expanded into the clinical setting, we chose to apply one data processing algorithm to all tasks. The reasoning for this decision was to apply an algorithm that was generalizable to tasks in different planes and, therefore, could be useful to those without advanced coding experience. Additionally, all sensors were filtered using a 6-Hz low pass Butterworth filter.

Turns at the head and trunk were calculated based on peaks in angular velocity data in the respective sensors. During the coin task, the pitch plane was assessed. During the jacket task, the yaw plane was assessed. Both yaw and pitch plane motions were assessed for the CAT. Initial processing involved identifying whether individual peaks in the angular rotation data constituted a turn. A peak was considered a turn if it had an amplitude (1) greater than 20°/s and (2) greater than 35% of the mean amplitude of all peaks for that participant. Once individual turns were identified, the direction, frequency, velocity, and amplitude were gathered for each head turn. The absolute values of each head turn’s velocity and amplitude were calculated, and overall means were calculated to represent the average peak amplitude and velocity of head turns occurring for each participant for each task. To determine the degree of the head and trunk coupling during the study tasks, correlation coefficients were also calculated between the head and trunk angular velocity signals. The Pearson correlation coefficient was defined as:

(1) $$\rho \left(A,\;B\right)=\frac{1}{N-1}\;\overset{N}{\underset{i=1}{\sum }}\left(\;\frac{A_{i}-\mu_{A}}{\sigma_{A}}\;\right)\left(\;\frac{B_{i}-\mu_{B}}{\sigma_{B}}\;\right)$$

*N* is the number of observations for each variable, and *µ* and *σ* are the mean and standard deviation, respectively. A high correlation of 1 represented a perfect match in the velocity signal and substantial coupling of head and trunk motion, while a low correlation of 0 represented a case where there was little coupling between the segments.

Based on previous research examining head movements in individuals post mild traumatic brain injury (mTBI) and controls [10], we selected peak head rotational velocity (degrees/s) as the primary outcome of interest. Secondary outcomes of interest were trunk rotational velocity(degrees/s), head and trunk amplitude of rotation (degrees), and head–trunk coupling correlation. Rotational velocity and amplitude of rotation were gathered in both the pitch and yaw planes. Head–trunk coupling (correlation between head and trunk velocities) was only calculated in the yaw plane. This measure was chosen as previous research has shown increased coupling of the head and trunk in the yaw plane with vestibular hypofunction [13]. To better characterize our participants, functional capacity was assessed with a distance covered during 2-min walk test (2MWT) [25].

### 2.4. Statistical Analysis

Paired *t*-tests were used to assess differences in outcomes between task conditions (with a cervical collar and without). Holm-Bonferroni correction was applied to the analysis of the primary outcome, head rotational velocity, for all tasks.

## 3. Results

Ten healthy participants consented and performed all tasks with and without a cervical collar. Participant demographic information is displayed in Table 1. The gait speed of each participant was within normal range per normative values [26]. There were no missing data, and all participants’ data was included in the analysis.

The results from the paired *t*-tests are listed in Table 2. Peak head rotational velocity was significantly decreased when wearing the collar in all but the coin task. Head–trunk coupling was significantly increased with the cervical collar application in all tasks (coin task *p* < 0.001; jacket task *p* = 0.007; and the CAT *p* = 0.003). Overall, there was an increase in trunk rotation amplitude with the addition of the cervical collar.

## 4. Discussion

As part of an effort to validate the utility of wearable sensors to accurately characterize limitations of head and trunk angular motions during daily life tasks that may be induced by vestibular injury or sensory integration deficits [10,13,20,21], we examined head and trunk kinematics during two conditions: a control condition with unconstrained cervical motion and a condition with constrained cervical motion. We utilized wearable sensors to quantify movements. As hypothesized, the suite of wearable sensors and data processing algorithms successfully distinguished between our control and constrained cervical motion condition on rotational velocity, amplitude, and head and trunk coupling.

### 4.1. Peak Turning Velocity

Peak head turn velocity was also significantly reduced during the jacket task (yaw plane) but not during the coin task (pitch plane). Such findings indicate that the IMU presents ecological utility in capturing differences in head turn velocity in some but not all ADL. This likely reflects the variability in task performance, as a task such as picking up the coin can be performed without isolated head movement and very little rotational velocity at the head (e.g., squatting rather than bending over). Similarly, Paul and colleagues [24] found a significant reduction in head rotation velocity during walking with directed horizontal head turns when participants wore a cervical collar compared to without. They demonstrated the sensitivity of the IMUs in measuring a rotation restriction created by the cervical collar during a pronounced stereotypical head turning task. Similar reductions in peak turning velocity have also been found in people following vestibular impairment during stereotyped turning tasks [13].

### 4.2. Head–Trunk Coupling

Head and trunk coupling significantly increased with the application of the collar (jacket *p* = 0.007, CAT *p* = 0.003) inferring that the IMUs were able to capture a common compensatory turning strategy, en bloc turning (head and body turning as one unit). Such results suggest that IMUs may be able to capture alterations in head–trunk coupling in individuals with varied types of vestibulopathies who often demonstrate alterations in head and trunk coordination during turning. Participants with a recent vestibular schwannoma resection increased head and trunk coupling compared to controls [13], while those with chronic mTBI symptoms performed turns with decreased head–trunk coupling [15].

### 4.3. Peak Turn Amplitude

The addition of the cervical collar to our participants decreased peak head turn amplitude during the CAT task, both in the pitch (head: *p* < 0.001) and yaw plane (head: *p* < 0.001, trunk: *p* = 0.005). However, unexpectedly, peak head turn amplitude increased with the collar during the coin task. This is possibly due to an alteration in the overall strategy to complete the task. Trunk rotation amplitude increased with the addition of the collar in yaw plane tasks (jacket and CAT), indicating that the IMUs were able to detect an increase in range of motion in the trunk to complete the task when cervical range of motion was limited. Aside from turning velocity, other turning metrics may be useful in distinguishing individuals with vestibular impairment from healthy controls [16,17]. Wang and colleagues [16] reported vestibular schwannoma patients generated similar head turning velocities to healthy controls; however, their head turns were greater in amplitude. Mijovic et al. [17] reported patients with a vestibular lesion utilized different strategies during ADL; some tasks were performed with faster rotational head velocities and other tasks were performed slower than the controls. These findings warrant further examination of an array of turning metrics in populations with turning difficulties to understand their compensatory turning strategies.

### 4.4. Limitations/Future Directions

There are several limitations in this study. First, the order of the turning tasks was not randomized. Second, the same data processing algorithm was applied to all tasks. Using the same algorithm was intended to simulate scenarios when head and trunk kinematics are assessed without advanced algorithm coding knowledge. However, the consequence of utilizing a generalizable algorithm is that we risk overlooking differences between conditions that a customized algorithm may detect. Lastly, despite the fact that we were adequately powered to see statistical differences between the conditions, the results of this study should be interpreted with caution considering the number of participants and their varied ages. Within this study, because of the functional relevance of yaw plane and pitch plane movements of the head to daily life, we constrained our analysis to these planes. In future studies, the use of IMUs provides the opportunity to calculate three-dimensional head kinematics as a means of characterizing deficits in head coordination during mobility tasks. In addition, future studies should examine refinement of data processing algorithms in capturing head and trunk kinematics in a variety of real-world scenarios (e.g., household chores, walking outside). This study provides a means to calculate appropriate sample size for head and trunk motion during ADL. Many studies have applied IMUs to capture gait and balance characteristics in populations with movement disorders with impaired turning mechanics [7,13,14,17,23,27]; however, capturing turning during ADL and meaningful tasks (e.g., dressing, preparing meals) in these populations without direct researcher observation would increase understanding of what daily activities lead to compensatory movements and challenge head and trunk control.

## 5. Conclusions

Wearable sensors are a feasible method of detecting the mechanical effects of cervical spine immobilization on rotational velocity, amplitude, and head and trunk coupling during ADL. These results complement existing research and provide the foundation for future investigations which quantify head and trunk kinematics in populations with vestibular and sensory integration impairments during real-world scenarios.

## Figures and Tables

**Table 1:** Demographic characteristics of healthy participants.

Characteristics (*n* = 10)	Mean ± Standard Deviation
Female/male	5/5
Age (years)	39.5 ± 12.47
Height (cm)	174.75 ± 9.37
Weight (kg)	75.02 ± 15.48
2MWT (m)	228.26 ± 15.23

**Table 2:** Mean, median standard deviation, and *t*-test results of rotational outcomes with and without cervical collar.

Turning Characteristics	Plane of Motion	No Collar		Collar	
**Head Rotational Velocity** **(Degrees/Second)**		**Mean**	**SD**	**Mean**	**SD**
Coin task	Pitch	110.63	22.27	122.09	25.86
Jacket task	Yaw	150.6	47.58	**112.1**	**20.63**
CAT	Pitch	83.18	10.08	**67.56**	**4.94**
CAT	Yaw	138.68	14.03	89.2	7.91
**Head Rotation Amplitude (degrees)**		**Mean**	**SD**	**Mean**	**SD**
Coin task	Pitch	41.18	10.87	**61.16**	**22.56**
Jacket task	Yaw	54.06	17.46	50.14	16.94
CAT	Pitch	17.74	3.5	**12.83**	**3.06**
CAT	Yaw	41.6	4.16	**28.78**	**4.85**
**Trunk Rotational Velocity (degrees/second)**		**Mean**	**SD**	**Mean**	**SD**
Coin task	Pitch	139.07	22.56	145.21	13.13
Jacket task	Yaw	94.43	18.48	107.46	20.74
CAT	Pitch	77.36	9.23	74.25	7.05
CAT	Yaw	88.31	8.92	89.62	8.23
**Trunk Rotation Amplitude (degrees)**		**Mean**	**SD**	**Mean**	**SD**
Coin	Pitch	72.92	17.40	77.12	17.36
Jacket	Yaw	37.22	14.94	**52**	13.3
CAT	Pitch	9.49	1.94	8.86	1.4
CAT	Yaw	25.65	2.27	**29.97**	4.81
Head–Trunk Correlation		CC mean/SD		CC mean/SD	
Coin	Pitch	0.65/0.13		0.87/0.12	
Jacket task	Yaw	0.66/0.12		**0.90**/0.13	
CAT	Yaw	0.52/0.05		**0.93**/0.03	

Mean and standard deviation (SD) for turning characteristics are listed for each task and the plane of motion. Significance level was set at 0.05. Significant changes in the collared task from no collar are bolded. CC = Correlation Coefficient.

## Data Availability

The data presented in this study are available on request.
